# The effectiveness of parathyroid gland autotransplantation in preserving parathyroid function during thyroid surgery for thyroid neoplasms: A meta-analysis

**DOI:** 10.1371/journal.pone.0221173

**Published:** 2019-08-14

**Authors:** Bin Wang, Chun-Rong Zhu, Hong Liu, Jian Wu

**Affiliations:** 1 Department of Thyroid and Breast Surgery, The Third People's Hospital of Chengdu, Chengdu, Sichuan, China; 2 Sichuan Key Laboratory of Medical Imaging, Department of Radiology, Affiliated Hospital of North Sichuan Medical College, Nanchong, Sichuan, China; 3 Department of Chemistry, School of Basic Medical Science, North Sichuan Medical College, Nanchong, China; University of Manitoba, CANADA

## Abstract

**Objective:**

We conducted this meta-analysis to assess the effectiveness of parathyroid gland autotransplantation in preserving parathyroid function during thyroid surgery for thyroid neoplasms.

**Methods:**

We conducted a search by using PubMed, Embase, and the Cochrane Library electronic databases for studies that were published up to January 2019. The reference lists of the retrieved articles were also reviewed. Two authors independently assessed the methodological quality and extracted the data. A random-effects model was used to calculate the overall combined risk estimates. Publication bias was evaluated with a funnel plot using Egger’s and Begg’s tests.

**Results:**

A total of 25 independent studies involving 10,531 participants were included in the meta-analysis. Compared with patients who did not undergo parathyroid gland autotransplantation, the overall pooled relative risks for patients who underwent parathyroid gland autotransplantation were 1.75 (95% CI: 1.51–2.02, p<0.001) for postoperative hypoparathyroidism, 1.72 (95% CI: 1.45–2.05, p<0.001) for protracted hypoparathyroidism, 1.06 (95% CI: 0.44–2.58, p = 0.894) and 0.71 (95% CI: 0.22–2.29, p = 0.561) for biochemical hypoparathyroidism and biochemical hypocalcemia at 6 months postoperatively, respectively, and 1.89 (95% CI: 1.33–2.69, p<0.001) and 0.22 (95% CI: 0.09–0.52, p = 0.001) for biochemical hypoparathyroidism and biochemical hypocalcemia at 12 months postoperatively, respectively. The pooled relative risks for patients who underwent one parathyroid gland autotransplantation and patients who underwent two or more parathyroid gland autotransplantations were 1.71 (95% CI: 1.25–2.35, p = 0.001) and 2.22 (95% CI: 1.43–3.45, p<0.001) for postoperative hypoparathyroidism, 1.09 (95% CI: 0.59–2.01, p = 0.781) and 0.55 (95% CI: 0.16–1.87, p = 0.341) for hypoparathyroidism at 6 months postoperatively compared with those of patients who did not undergo parathyroid gland autotransplantation.

**Conclusions:**

Parathyroid gland autotransplantation is significantly associated with increased risk of postoperative and protracted hypoparathyroidism, and the number of autoplastic parathyroid glands is positively correlated with the incidence of postoperative hypoparathyroidism.

## Introduction

The incidence of thyroid carcinoma has been increasing globally for a variety of reasons[1–8]. As the primary therapy method for thyroid carcinoma, surgery is associated with some complications. Hypoparathyroidism is one of the main complications. The incidence of transient hypoparathyroidism was 17%-55.7%, and the incidence of permanent hypoparathyroidism varied from 0 to 16.2% across different studies[9–11]. Hypoparathyroidism was the result of injury to the parathyroid glands from trauma, devascularization, or unintentional removal[12, 13]. This complication may prolong hospitalization, lead to readmission and increase the overall costs of thyroid surgery[14–16].

Parathyroid gland autotransplantation(PGA) has been widely used to preserve parathyroid function in recent decades[16–21]. Some researchers have advocated routine PGA to protect its function[22–24]. However, some studies have demonstrated that PGA increases the risk of postoperative hypoparathyroidism[25–28]. Some studies have reported that PGA increases the incidence of transient hypoparathyroidism but does not affect the incidence of permanent hypoparathyroidism[16, 29–31]. Although Iorio and his colleague[32] conducted a systematic review that focused on the aspects of the PGA procedure, indications, technique and results, these researchers only summarized and described the studies and did not perform a statistical analysis. In general, there is still no agreement on the relationship between hypoparathyroidism and PGA. Therefore, we conducted this meta-analysis to assess the effectiveness of PGA in preserving parathyroid function.

## Methods

### Search strategy

We attempted to follow the proposed Meta-Analysis of Observational Studies in Epidemiology (MOOSE) guidelines[33] to conduct the present meta-analysis. We conducted a search by using PubMed, Embase, and the Cochrane Library electronic databases for studies that were published up to January 2019. The following search terms were used in all fields as a search strategy: 1) transplantation, autologous, autograft, autografts, autologous transplantation, autologous transplantations, (Transplantations, Autologous), autotransplant, autotransplants, autotransplantation, autotransplantations, autografting, and autograftings; 2) parathyroid glands, parathyroid gland, (gland, parathyroid), (glands, parathyroid), and parathyroid; 3) thyroidectomy, thyroidectomies, thyroid gland excision, excision of thyroid gland, surgery, surgeries, operation, operations, (surgical procedures, operative), operative surgical procedure, operative surgical procedures, (procedures, operative surgical), (surgical procedure, operative), operative procedures, operative procedure, (procedure, operative), (procedures, operative), (procedure, operative surgical), and operative therapy; and 4) thyroid neoplasms, (neoplasm, thyroid), thyroid neoplasm, (neoplasms, thyroid), thyroid carcinoma, (carcinoma, thyroid), (carcinomas, thyroid), thyroid carcinomas, cancer of thyroid, thyroid cancers, thyroid cancer, (cancer, thyroid), (cancers, thyroid), cancer of the thyroid, thyroid adenoma, (adenoma, thyroid), (adenomas, thyroid), and thyroid adenomas. No restrictions were imposed. In addition, we reviewed the reference lists of the retrieved papers and recent reviews.

### Study selection

We first performed an initial screening of the titles and abstracts. A second screening was performed based on the full-text review. Studies were considered eligible if they met the following criteria: 1) the study was published in English; 2) the thyroid surgery in the study was the initial surgery; 3) the minimal scope of the surgery in the study was near-total thyroidectomy; 4) the exposure of interest included PGA; 5) the outcome of interest was the incidence of hypoparathyroidism and/or hypocalcemia; and 6) relative risk (RR) and the corresponding 95% confidence interval (CI) (or data to calculate these values) were reported. Studies were excluded based on the following criteria: 1) those including thyroidectomy for multiple endocrine neoplasia; 2) those including thyroidectomy for parathyroid adenoma, coincident with thyroid carcinoma or not; and 3) those in which the full text of the studies could not be accessed online or by request to the authors.

### Data extraction and quality assessment

Data extraction was then performed by using a standardized data-collection form. Data were collected as follows: the first author, year of publication, type of study, country of origin, study period, duration of follow-up, sample size and the number of cases and controls, surgical approach, method and site of PGA, number of patients with different numbers of autoplastic parathyroid glands, evaluation indexes of hypoparathyroidism and hypocalcemia, number of patients with hypoparathyroidism or hypocalcemia after surgery, and RR and the corresponding 95% confidence interval. The quality of retrospective and prospective cohort studies and case-control studies was assessed with the Newcastle-Ottawa Scale (NOS)[34]. The studies with an NOS score ≥6 were considered high-quality studies. Two authors (WANG B and ZHU CR) independently conducted the study selection, data extraction, and quality assessment. All disagreements in these processes were discussed and resolved by consensus.

### Statistical analyses

Pooled RR was used as a measure of the association between the function of parathyroid glands and PGA across studies. Heterogeneity was quantified statistically with the I^2^ test. P < 0.1 and I^2^ > 50% for heterogeneity were considered significant differences. A random-effects model (DerSimonian-Laird) was used to calculate the pooled RRs for all analyses. If there was heterogeneity, subgroup analysis was conducted according to the different evaluation indexes of hypoparathyroidism. Potential publication bias was assessed by visual inspection of the Egger funnel plots, in which the log RRs were plotted against their SEs. We also performed the Begg rank correlation test[35] and the Egger linear regression test[36] at the p < 0.05 level of significance. All analyses were performed using Stata version 14.0 (Stata Corp LP, College Station, Texas, USA). P < 0.05 was considered statistically significant in all tests.

## Results

### Literature search

**Fig 1:**
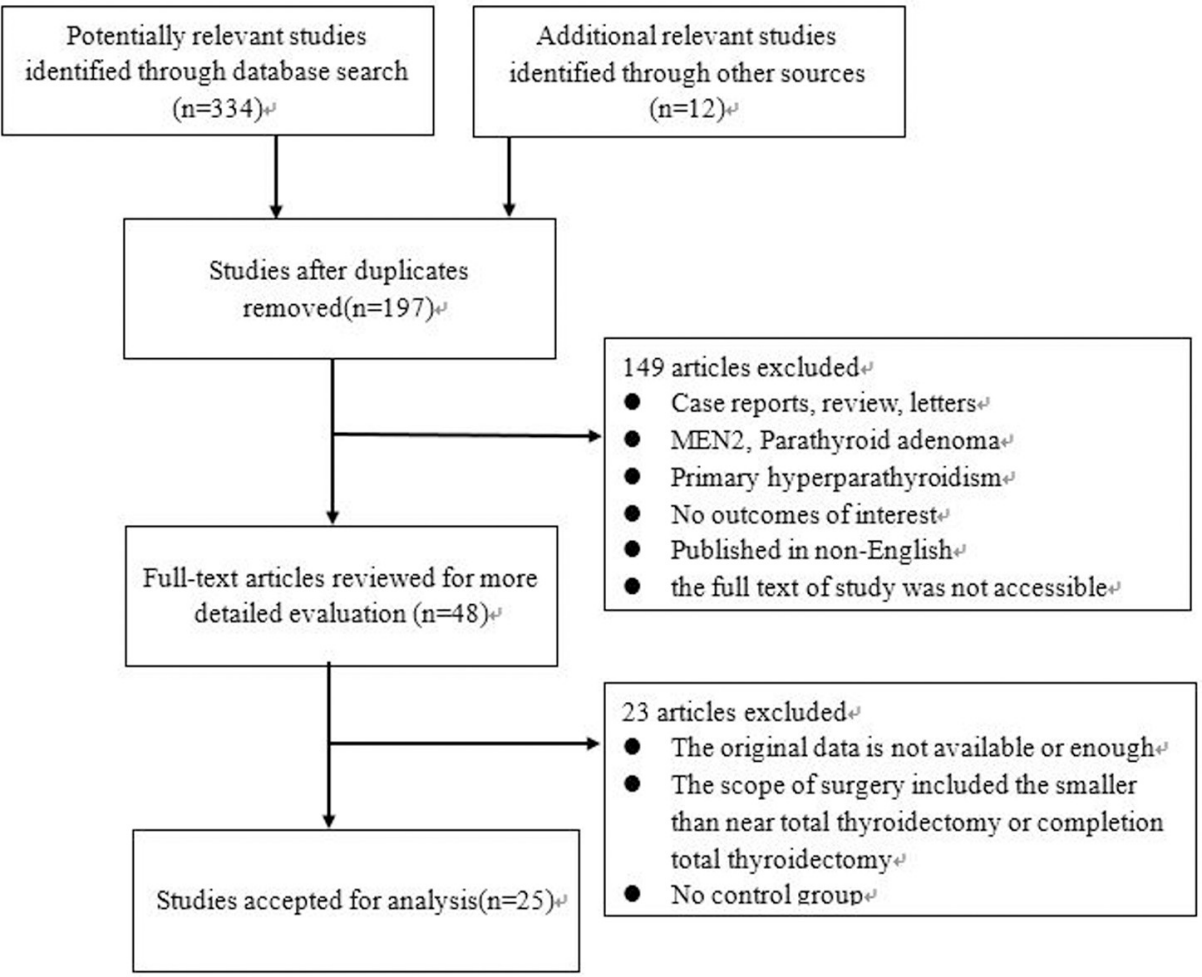
Flow chart of study selection.

The study selection process is shown in Fig 1. Through searching the databases, a total of 346 potentially relevant records were identified, 197 of which were retained after duplicates were removed. After screening the titles and abstracts, 149 studies were excluded for various reasons. The remaining 48 studies were assessed for eligibility via full-text screening, and 23 studies were further excluded. Finally, 25 independent studies were included in the meta-analysis.

### Study characteristics

Table 1 summarizes the basic information of the 25 included eligible studies[9, 10, 16, 19–21, 24, 26–31, 37–48]. These studies were published between 1977 and 2018. Among the 25 studies, there was only one prospective cohort study[19], and the other studies were retrospective cohort studies or case-control studies[9, 10, 16, 20, 21, 24, 26–31, 37–48]. Of the 25 cohort studies, 15 were conducted in Asia, 6 in Europe, 2 in the United States, and 2 in Australia. The sample size of the studies varied from 65 to 1196, and the total study population was 10,531 participants. One study[42] that described the completion of total thyroidectomy was also included because of the detailed information regarding the initial surgery in the study. The evaluation indexes of hypoparathyroidism varied across studies, including biochemical hypoparathyroidism, biochemical hypocalcemia and symptomatic hypocalcemia (the presence of the symptoms of hypocalcemia). The evaluation time ranged from 1 day postoperatively to 12 months postoperatively. Postoperative, protracted and permanent hypoparathyroidism was defined as when the evaluation indexes qualified as hypoparathyroidism when they were measured 1 month postoperatively, 1 month to 6 months postoperatively, and not less than 6 months postoperatively, respectively. According to the NOS, all the included studies demonstrated relatively high quality, with the distribution of the scores of these studies ranged from six to eight.

**Table 1:** Basic characteristics of the included studies.

authors	year	study design	country	duration	sample size	age (yeas old)	follow-up time (months)	surgical method	sites	evaluation times	evaluation indexes
Salander et al[19].	1977	PC	Sweden	1972.1–1976.4	97	NA	6–60	TT	SCM or AM of the thigh.	6m	biochemical hypocalcemia
Gann et al[37].	1979	RC	American	1973–1978	71	NA	6	TT	SCM	1d,2m	biochemical hypocalcemia
Kikumori et al[38].	1999	RCC	Japan	1992.1–1996.12	104	NA	34	TT, TT+BCND	PMM	1m	biochemical hypoparathyroidism, biochemical hypocalcemia
Palazzo et al[16].	2005	RC	Australia	1998.7–2003.6	1196	NA	6	TT	NA	1d,6m	biochemical hypocalcemia
Abboud et al[9].	2008	RCC	Lebanon	2002.1–2005.6	252	36–55	12–72	TT	ISCM	1d	biochemical hypocalcemia
Ebrahimi et al[26].	2009	RC	Australia	2004–2005	628	NA	6	TT	SCM	1d,6m	biochemical hypocalcemia
Sokouti et al[40].	2010	RCC	Iran	2002–2006	65	40.6±10.8	6	TT, TT+UCND	DM or SCM	1d	biochemical hypocalcemia
Sitges-Serra et al[39].	2010	RCC	Spain	1993–2007	425	56±15	12	TT, TT+CND, TT+CND+LND	ISCM	1d,1m,12m	biochemical hypocalcemia, biochemical hypoparathyroidism
Ahmed et al[24].	2013	RC	Pakistan	1998.7–2009.6	388	NA	6	TT	ISCM	1m,6m	biochemical hypocalcemia
Paek et al[41].	2013	RCC	Republic of Korea	2003.3–2006.8	531	NA	12	TT, TT+UCND, TT+BCND	SCM	2d,12m	semiotic hypocalcemia
Ito et al[42].	2014	RC	Japan	2005.2–2012.6	154	NA	12	TT, TT+UCND, TT+BCND	CSCM	12m	biochemical hypocalcemia
Wei et al[20].	2014	RC	China	2007.2–2012.2	477	14–72	23–81	TT+BCND	CSCM	1d,6m	biochemical hypoparathyroidism
Lorente-Poch et al[28].	2015	RC	Spain	1998–2012	657	12–86	12	TT, TT+CND, TT+CND+LND	ISCM	1d,1m,12m	biochemical hypocalcemia, biochemical hypoparathyroidism
Uruno et al[45].	2016	RC	Japan	2012.10–2014.9	411	NA	12	TT+UCND	NA	12m	biochemical hypocalcemia
White et al[27].	2016	RC	American	2012.7–2013.12	196	10–82	0.5	TT, TT+UCND	ISCM	2weeks	semiotic hypocalcemia
Tartaglia et al[44].	2016	RC	Italy	2001.1–2010.12	244	NA	6	TT	ISCM	1d,6m	biochemical hypoparathyroidism, biochemical hypocalcemia
Lang et al[43].	2016	RCC	China	2010–2013	569	52.6 ± 14.2	12	TT	SCM	1d,1m,12m	biochemical hypocalcemia, biochemical hypoparathyroidism
Sonne-Holm et al[29].	2017	RCC	Denmark	2010.1–2015.3	575	11–95	12	TT, TT+UCND	SCM	1d,3m,12m	biochemical hypoparathyroidism
Su et al[30].	2017	RCC	China	2013.1–2016.6	903	43.2±13.9	6	TT+UCND, TT+BCND	CSCM	1d,6m	biochemical hypoparathyroidism
Kirdak et al[10].	2017	RC	Turkey	2007.1–2015.12	122	19–71	6	TT	SCM	1d,6m	biochemical hypoparathyroidism, biochemical hypocalcemia
Fama et al[46].	2017	RC	Italy	2013.1–2014.12	396	NA	12	TT	SCM	1d,12m	biochemical hypocalcemia, semiotic hypocalcemia
Su et al[31].	2018	RC	China	2012–2015	766	19–80	24	TT+UCND, TT+BCND	CSCM	1d,6m	biochemical hypoparathyroidism
Su et al[21].	2018	RC	China	2013.1–2016.6	702	42.6±12.9	6	TT+UCND, TT+BCND	CSCM	1d,6m	biochemical hypoparathyroidism
Teshima et al[48].	2018	RCC	Japan	2012–2017	65	17–86	6	TT+UCND	SCM	1d,6m	semiotic hypocalcemia
Su et al[47].	2018	RC	China	2014.11–2016.11	537	17–72	6	TT+BCND	SCM	6m	biochemical hypoparathyroidism

PC prospective cohort study, RC retrospective cohort study, RCC retrospective case-control study, NA not acknowledge, TT total thyroidectomy, CND central lymph node dissection, UCND unilateral central lymph node dissection, BCND bilateral central lymph node dissection, SCM sternocleidomastoid muscle, ISCM ipsilateral sternocleidomastoid muscle, CSCM contralateral sternocleidomastoid muscle, AM adductor muscles, DM deltoid muscle, PMM pectoralis major muscle.

### PGA and the risk of postoperative hypoparathyroidism

**Fig 2:**
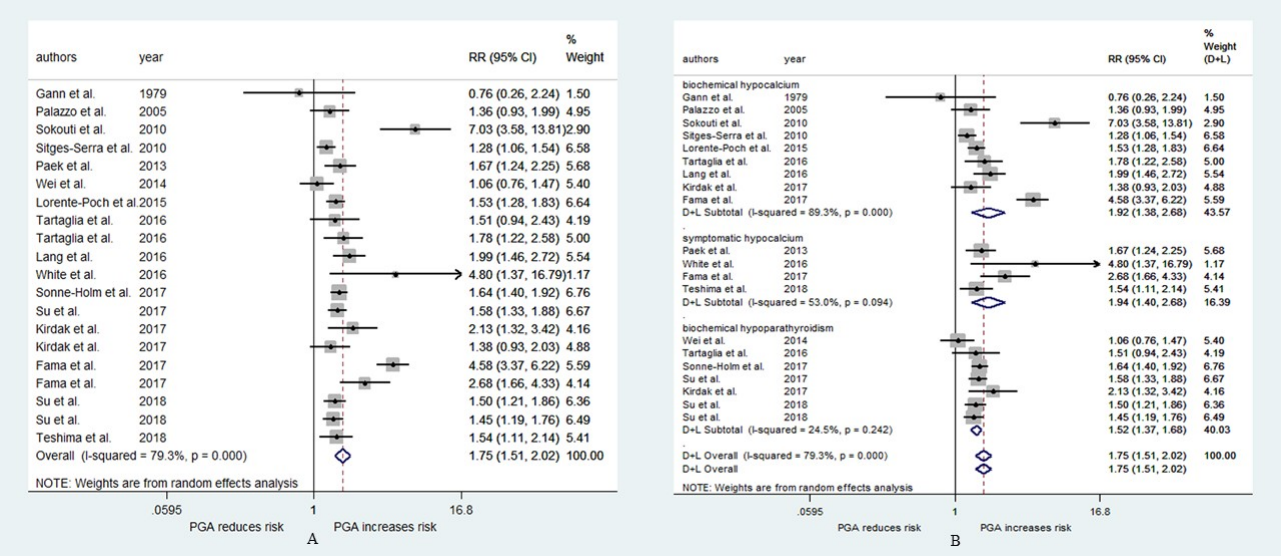
RR for postoperative hypoparathyroidism in patients who underwent PGA vs patients who not underwent PGA. (A) overall relative risks. (B) the relative risk in subgroup of different evaluation indexes.

There were 17 studies[10, 16, 20, 21, 27–31, 37, 39–41, 43, 44, 46, 48] that explored the relationship between PGA and the risk of postoperative hypoparathyroidism. Among them, 3 studies[10, 44, 46] used two different evaluation indexes. Significant heterogeneity was detected (I^2^ = 79.3%, p<0.001). The pooled RR from all of these studies was 1.75 (95% CI: 1.51–2.02, p<0.001, Fig 2A), and the publication bias as measured by Begg’s and Egger’s tests did not appear to be significant (p = 0.183, p = 0.138). To address the heterogeneity, we performed a subgroup analysis according to the different evaluation indexes. Weak heterogeneity was observed in the biochemical hypoparathyroidism subgroups, and strong heterogeneity was observed in the biochemical hypocalcemia and symptomatic hypocalcemia subgroups (Fig 2B). The pooled RRs for the biochemical hypoparathyroidism, biochemical hypocalcemia and symptomatic hypocalcemia subgroups were 1.52 (95% CI: 1.37–1.68, p<0.001), 1.92 (95% CI: 1.38–2.68, p<0.001), and 1.94 (95% CI: 1.40–2.68, p<0.001), respectively (Fig 2B).

### PGA and the risk of protracted hypoparathyroidism

**Fig 3:**
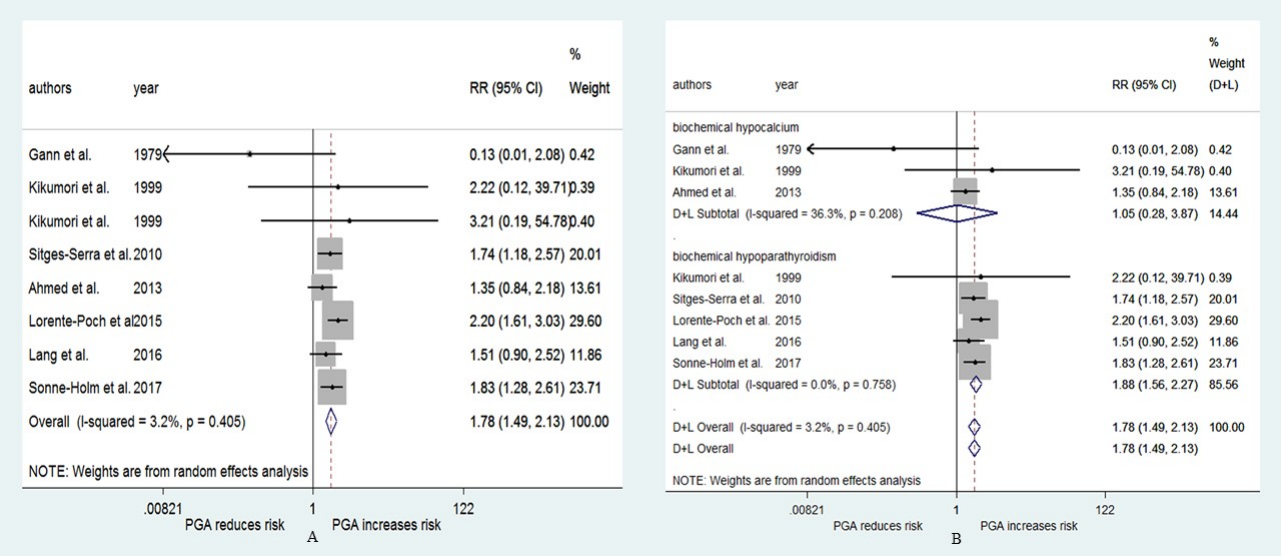
RR for protracted hypoparathyroidism in patients who underwent PGA vs patients who not underwent PGA. (A) overall relative risks. (B) the relative risk in subgroup of different evaluation indexes.

Fig 3A shows the result of the pooled RR for the risk of protracted hypoparathyroidism. Seven studies[24, 28, 29, 37–39, 43] were included in the analysis, of which 1 study[38] used two evaluation indexes. The RRs for the association varied from 0.13 to 3.21 across the studies, while the pooled RR was 1.78 (95% CI: 1.49–2.13, p<0.001, Fig 3A). The heterogeneity was weak (I^2^ = 3.2%, p = 0.405), and the publication bias was not significant (Begg, p = 0.536; Egger, p = 0.277). The pooled RR in relation to PGA was 1.88 (95% CI: 1.56–2.27, p<0.001, Fig 3B) for biochemical hypoparathyroidism and 1.05 (95% CI: 0.28–3.87, p = 0.945, Fig 3B) for biochemical hypocalcemia. No heterogeneity was observed in the biochemical hypoparathyroidism subgroup (Fig 3B).

### PGA and the risk of permanent hypoparathyroidism

**Fig 4:**
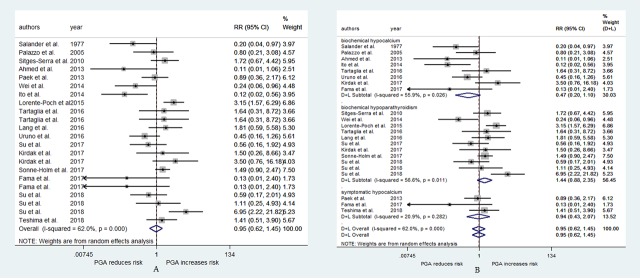
RR for permanent hypoparathyroidism in patients who underwent PGA vs patients who not underwent PGA. (A) overall relative risks. (B) the relative risk in subgroup of different evaluation indexes.

The result that is presented in Fig 4A combines the RRs for the risk of permanent hypoparathyroidism. Nineteen studies[10, 16, 19–21, 24, 28–31, 39, 41–48] were used to generate the result, of which 3 studies[10, 44, 46] used two evaluation indexes. PGA was not associated with the risk of permanent hypoparathyroidism (RR = 0.95, 95% CI: 0.62–1.45, p = 0.801, Fig 4A), and substantial heterogeneity was observed (I^2^ = 62.0%, p<0.001, Fig 4A). After performing the subgroup analysis, we found the same result that PGA was not related to the risk of permanent hypoparathyroidism (biochemical hypoparathyroidism RR = 1.44, 95% CI: 0.88–2.35, p = 0.15; biochemical hypocalcemia RR = 0.47, 95% CI: 0.20–1.10, p = 0.08; symptomatic hypocalcemia RR = 0.94, 95% CI: 0.43–2.07, p = 0.887; Fig 4B). The heterogeneities were both statistically significant in the biochemical hypoparathyroidism subgroup and in the biochemical hypocalcemia subgroup (Fig 4B). The publication bias was also statistically significant (Begg, p = 0.048; Egger, p = 0.02).

**Fig 5:**
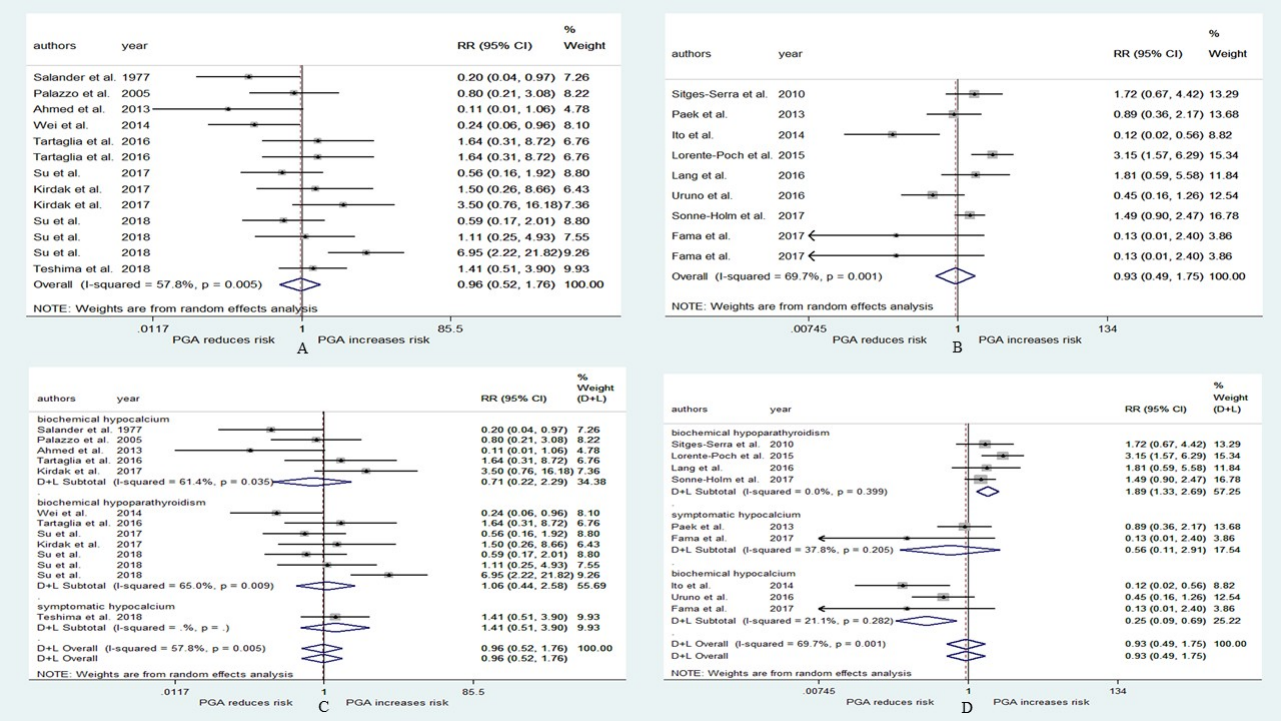
RR for hypoparathyroidism at postoperative 6 and 12 months in patients who underwent PGA vs patients who not underwent PGA. (A) overall relative risks at postoperative 6 months. (B) overall relative risks at postoperative 12 months. (C) the relative risk in subgroup of different evaluation indexes at postoperative 6 months. (D) the relative risk in subgroup of different evaluation indexes at postoperative 12 months.

Because permanent hypoparathyroidism consisted of the evaluation indexes at 6 months and 12 months postoperatively, the pooled RRs for the risk of hypoparathyroidism are displayed in Fig 5 according to the time of evaluation. The pooled RRs for hypoparathyroidism at 6 months and 12 months postoperatively were 0.96 (95% CI: 0.52–1.76, p = 0.886; Begg, p = 0.625; Egger, p = 0.287; Fig 5A) and 0.93 (95% CI: 0.49–1.75, p = 0.816; Begg, p = 0.061; Egger, p = 0.042; Fig 5B). The pooled RRs for biochemical hypoparathyroidism, biochemical hypocalcemia, and symptomatic hypocalcemia were 1.06 (95% CI: 0.44–2.58, p = 0.894, Fig 5C), 0.71 (95% CI: 0.22–2.29, p = 0.561, Fig 5C), and 1.41 (95% CI: 0.51–3.90, p = 0.511, Fig 5C) at 6 months postoperatively, 1.89 (95% CI: 1.33–2.69, p<0.001, Fig 5D), 0.25 (95% CI: 0.09–0.69, p = 0.008, Fig 5D), and 0.56 (95% CI: 0.11–2.91, p = 0.49, Fig 5D) at 12 months postoperatively.

### The number of autoplastic parathyroid glands and the risk of hypoparathyroidism

**Fig 6:**
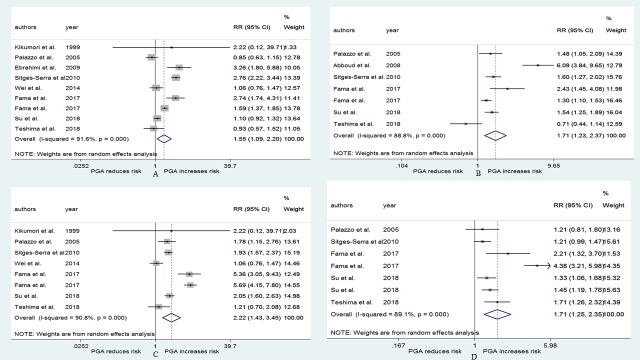
RR for postoperative hypoparathyroidism in patients who underwent different number of PGA. (A) two or more vs one or less. (B) two or more vs one. (C) two or more vs zero. (D) one vs zero.

Fig 6 presents the pooled RRs related to the different number of autoplastic parathyroid glands and the risk of postoperative hypoparathyroidism. The incidence of postoperative hypoparathyroidism in the group where the patients underwent two or more parathyroid gland autotransplantations was higher than that in the group where the patients underwent one or fewer parathyroid gland autotransplantations (RR = 1.55, 95% CI: 1.09–2.20, p = 0.014; Begg, p = 0.602; Egger, p = 0.863; Fig 6A), that in the group where the patients underwent 1 parathyroid gland autotransplantation (RR = 1.71, 95% CI: 1.23–2.37, p = 0.001; Begg, p = 0.548; Egger, p = 0.371; Fig 6B), and that in the group where the patients did not undergo parathyroid gland autotransplantation (RR = 2.22, 95% CI: 1.43–3.45, p<0.001; Begg, p = 0.711; Egger, p = 0.861; Fig 6C). The incidence of postoperative hypoparathyroidism in the group where the patients underwent only 1 parathyroid gland autotransplantation was also higher than that in the group where the patients did not undergo autotransplantation (RR = 1.71, 95% CI: 1.25–2.35, p = 0.001; Begg, p = 0.23; Egger, p = 0.328; Fig 6D).

**Fig 7:**
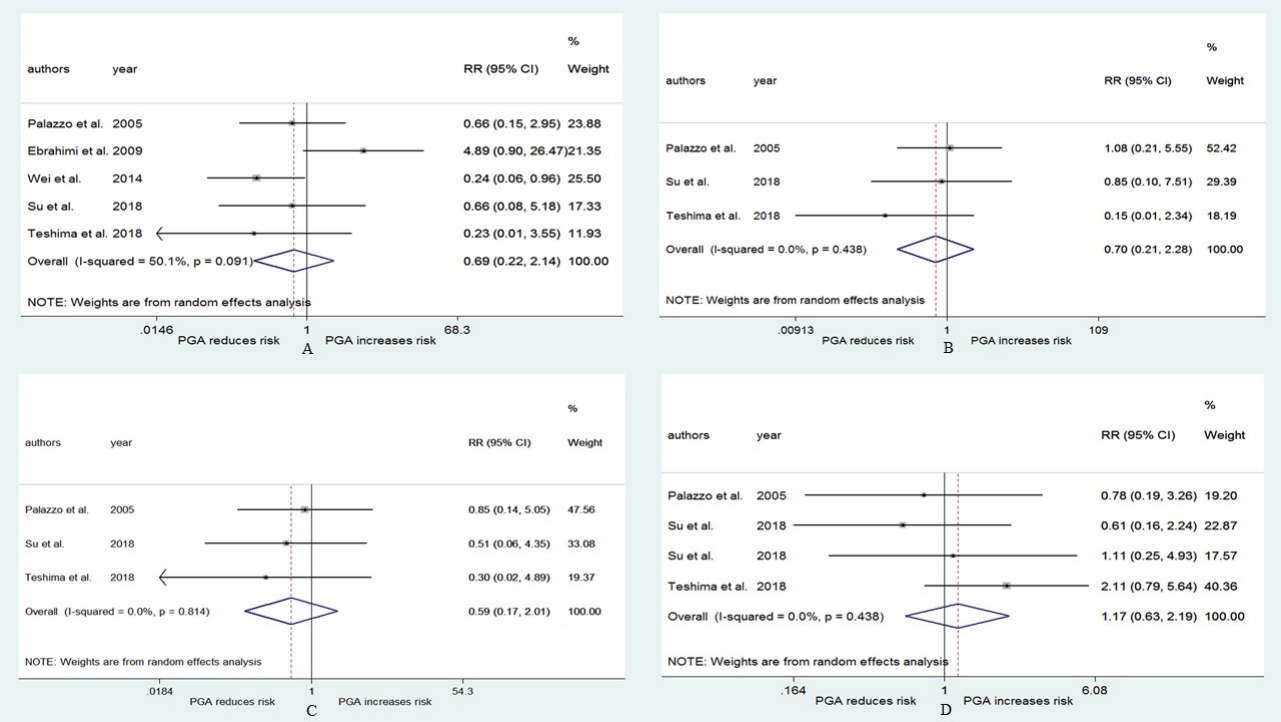
RR for permanent hypoparathyroidism in patients who underwent different number of PGA. (A) two or more vs one or less. (B) two or more vs one. (C) two or more vs zero. (D) one vs zero.

Compared with the group in which patients underwent two or more parathyroid gland autotransplantations, the incidence of permanent hypoparathyroidism was not significantly different in the group in which the patients underwent one or fewer parathyroid gland autotransplantations (RR = 0.69, 95% CI: 0.22–2.14, p = 0.523; Begg, p = 0.806; Egger, p = 0.967; Fig 7A), that in the group in which the patients underwent 1 parathyroid gland autotransplantation (RR = 0.7, 95% CI: 0.21–2.28, p = 0.55; Begg, p = 0.296; Egger, p = 0.278; Fig 7B), and that in the group in which the patients did not undergo parathyroid gland autotransplantation (RR = 0.59, 95% CI: 0.17–2.01, p = 0.398; Begg, p = 0.296; Egger, p = 0.103; Fig 7C). No significant difference in permanent hypoparathyroidism was observed between the group in which patients underwent only 1 parathyroid gland autotransplantation and in the group in which the patients did not undergo autotransplantation (RR = 1.17, 95% CI: 0.63–2.19, p = 0.617; Begg, p>0.999; Egger, p = 0.207; Fig 7D).

## Discussion

PGA was first performed in humans by Lahey in 1926[49] and has become the most common method to preserve the function of the parathyroid gland, which cannot be preserved at its site[16, 41, 46]. In recent years, some surgeons have suggested that PGA should be routinely performed during total thyroidectomy[22–24]. However, there is not adequate evidence for this idea, and it is still unclear whether PGA increases or reduces the incidence of permanent hypoparathyroidism[16, 30, 31, 40, 43, 44]. Therefore, a meta-analysis was conducted to address the controversy to explore the relationship between -PGA and the risk of hypoparathyroidism.

According to this meta-analysis, PGA was associated with increased risk of postoperative hypoparathyroidism. Although the heterogeneity was significant, most of the studies showed similar results[21, 27–31, 39–41, 43, 48]. When the evaluation indexes were measured within one month, especially at 24 hours postoperatively, the function of the autoplastic parathyroid gland had not recovered, and the parathyroid gland(s) at its site contributed to almost all of the function at the time, so the number of functional parathyroid glands was lower in the group in which the patients underwent PGA than that in the group in which the patients did not undergo PGA. When pooling the RR for the different evaluation indexes to reduce the heterogeneity, we found that the results were consistent with the overall results, especially the results from biochemical hypoparathyroidism, a direct index of parathyroid dysfunction, the heterogeneity for which was no longer statistically significant.

In terms of protracted hypoparathyroidism, which may become a permanent complication, we also confirmed that PGA was associated with increasedrisk. The parathyroid glands at their sites and the autoplastic parathyroid glands both had an effect on the parathyroid function at the time, so the relationship between PGA and the risk of protracted hypoparathyroidism was unclear[24, 28, 29, 37–39, 43]. However, we found that the heterogeneity was small across these studies and became zero in the biochemical hypoparathyroidism subgroup when pooling the RR for the risk of protracted hypoparathyroidism. Some studies[50–52] have confirmed that it takes 4 to 14 weeks for the grafted parathyroid to recover function. The phenomenon might be attributed to the speculation that the autoplastic parathyroid glands did not fully function, especially because the evaluation indexes were measured at 1 month postoperatively in most of the included studies[24, 28, 38, 39, 43].

Regarding permanent hypoparathyroidism, some researchers believed that PGA increased its risk because of the discovery that the autoplastic parathyroid could not completely recover function[25, 53, 54], and this meta-analysis showed no significant effect from PGA. The same results were obtained in the different evaluation index subgroups. However, the publication bias was statistically significant. We pooled the RRs in the subgroups according to the evaluation time and found that PGA had no effect on the risk of hypoparathyroidism at 6 months and 12 months postoperatively. In addition, publication bias was no longer significant in the subgroup analysis at 6 months postoperatively. We obtained the same results in the different evaluation index subgroups at 6 months postoperatively. However, when combining the RRs at 12 months postoperatively according to the different evaluation indexes, we obtained different and interesting results that showed that PGA increased the risk of biochemical hypoparathyroidism but reduced the risk of biochemical hypocalcemia and had no influence on the risk of symptomatic hypoparathyroidism. The autoplastic parathyroid glands were fully functioning at 6 months postoperatively, which might explain why the risk of hypoparathyroidism at that time from PGA was reduced compared to that at other time points. Regarding our observation of the opposite effect of PGA on biochemical hypoparathyroidism and hypocalcemia at 12 months postoperatively, the explanation[21] that various injuries, such as trauma, the loss of some tissue, and hematoma in the receptor site, led to the autoplastic parathyroid gland not functioning as well as it had before it was transplanted might be not applicable. The autoplastic parathyroid gland fibrosis and compensation might be responsible for this phenomenon. The parathyroid glands at their sites gradually recovered from surgical trauma and survived, while the autoplastic parathyroid survived and underwent fibrosis. The latter might be more beneficial for the occurrence of compensation[55, 56], which could lead to hypoparathyroidism but to normal serum calcium. Due to the small statistical differences, the publication bias might also be responsible for the observation of opposite effects.

Some studies have indicated that the incidence of permanent hypoparathyroidism is correlated with the number of autoplastic parathyroid glands. Several studies[9, 16, 20, 24, 40, 47] have revealed that the autotransplantation of one or more parathyroid glands could effectively reduce the incidence of permanent hypoparathyroidism, and Teshima and coworkers[48] confirmed that the autotransplantation of two or more parathyroid glands could prevent permanent hypoparathyroidism. This meta-analysis showed that when more parathyroid glands underwent autotransplantation, the incidence of postoperative hypoparathyroidism was higher. This was the result of the decrease of parathyroid glands at the site that play a key role in postoperative hypoparathyroidism. The number of autoplastic parathyroid glands had no connection to the incidence of permanent hypoparathyroidism. The included evaluation indexes were all measured at 6 months postoperatively, when the autoplastic parathyroid glands were fully functioning.

Substantial heterogeneity was observed among the studies regarding the relationship between PGA and the risk of postoperative, protracted, and permanent hypoparathyroidism, which was a major problem that affected the reliability of the pooled-effect size in the meta-analysis. The results of the subgroup analysis according to the different evaluation indexes of hypoparathyroidism showed that the heterogeneity was much smaller in some subgroups, but that the heterogeneity was high in other groups, suggesting that some other factors served as the sources of heterogeneity. The following factors might have influenced the heterogeneity: 1) diverse methods of serum parathyroid hormone and calcium detection were used; 2) the criteria of biochemical hypoparathyroidism and hypocalcemia in each study were not completely consistent; 3) the characteristics of the populations varied in the different studies; 4) the confounding factors were different across these studies, and some studies did not adjust these factors; and 5) the quality of each study (NOS score) was not completely consistent.

There are several limitations to this meta-analysis. First, because all included studies were cohort studies or case-control studies and high-quality randomized controlled trials had not be conducted, bias was inevitable. Second, the heterogeneity was still significant in some subgroups after the subgroup analysis was performed. Third, although little evidence of publication bias was observed in most of the groups, publication bias was observed in the permanent hypoparathyroidism group and was borderline in the subgroup that was at 12 months postoperatively. Finally, the reason for the observations of the opposite effect of PGA on biochemical hypoparathyroidism and on hypocalcemia at 12 months postoperatively was not completely clear.

## Conclusions

This meta-analysis suggests that PGA was significantly associated with increased risk of postoperative and protracted hypoparathyroidism. Due to the different effects on the risk of hypoparathyroidism at 6 months and 12 months postoperatively and the opposite influence on biochemical hypoparathyroidism and hypocalcemia at 12 months postoperatively, the relationship between PGA and permanent hypoparathyroidism is unclear. And the evidence for routine PGA is not abundant. Considering the limitations of our meta-analysis, further studies are needed to validate and to perfect these findings.

## Supporting information

S1 Table PRISMA checklist. (DOC)

S1 Fig PRISMA flow diagram. (DOC)
