# Supplementary material for: The effectiveness of parathyroid gland autotransplantation in preserving parathyroid function during thyroid surgery for thyroid neoplasms: A meta-analysis
Source: PLoS One. 2019 Aug 14;14(8):e0221173. doi: 10.1371/journal.pone.0221173 (PMC6693848; doi:10.1371/journal.pone.0221173)
Supplement: S1 Fig — (DOC) [file pone.0221173.s002.doc]

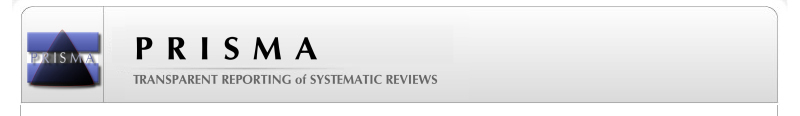
**PRISMA 2009 Flow Diagram**

**Screening**

**Included**

**Eligibility**

**Identification**

Records identified through database searching
(n = 334 )

Additional records identified through other sources
(n = 12 )

Records after duplicates removed
(n = 197 )

Records screened
(n = 197 )

Records excluded
(n =149 )

Full-text articles assessed for eligibility
(n = 48 )

Full-text articles excluded, with reasons
(n = 23 )

Studies included in qualitative synthesis
(n =25 )

Studies included in quantitative synthesis (meta-analysis)
(n = 25 )

**Table 1. Data for calculation of kappa statistic**

|  |  | **Review author 2** | | |  |
| --- | --- | --- | --- | --- | --- |
|  |  | Include | Exclude | Unsure | Total |
| **Review author 1** | Include | 24 | 0 | 0 | 24 |
|  | Exclude | 0 | 168 | 2 | 170 |
|  | Unsure | 1 | 0 | 2 | 3 |
|  | Total | 25 | 168 | 4 | 197 |

Kappa=0.94
